# A Primary Pulmonary Meningioma That Grew Over 10 Years: A Surgical Case Report

**DOI:** 10.7759/cureus.55204

**Published:** 2024-02-29

**Authors:** Somei Matsuo, Naoki Kanauchi

**Affiliations:** 1 Thoracic Surgery, Nihonkai General Hospital, Sakata, JPN

**Keywords:** lobectomy, positron emission tomography computed tomography, benign lung tumor, ectopic meningioma, primary pulmonary meningioma

## Abstract

Primary pulmonary meningiomas (PPMs) are rare meningothelial proliferation that lacks characteristic imaging findings, making their distinction from other peripheral lung tumors challenging. Therefore, surgical resection is often performed for the diagnosis and treatment of PPM. Herein, we describe a surgical case of PPM that grew over 10 years. A 63-year-old woman was referred to our department due to right middle lobe lung tumor enlargement. No significant symptoms were observed. Chest computed tomography revealed a tumor in the middle lobe of the right lung. F-18 fluorodeoxyglucose positron emission tomography showed accumulation in the nodule; thus, lung cancer could not be ruled out. Therefore, the preoperative differential diagnosis was cStageIB lung cancer. A right middle lobectomy was performed, and a histopathology examination revealed meningioma. There were no primary lesions in the head and whole spine magnetic resonance imaging, thus, a final diagnosis of PPM was made. Cautious observation is required postoperatively due to the possibility of recurrence.

## Introduction

Most meningiomas occur in the central nervous system, however, some can occur ectopically in the head, neck sites, and skin. Primary pulmonary meningiomas (PPM) are a rare type of ectopic meningiomas and account for 2% of all meningiomas [1]. Due to a lack of characteristic imaging findings of PPMs and the difficulty in making a definitive preoperative diagnosis, surgical resection is performed in most cases for definitive diagnosis and treatment. Herein, we describe the surgical resection of a PPM that was preoperatively suspected to be a lung cancer because of its growth.

## Case presentation

**Figure 1:**
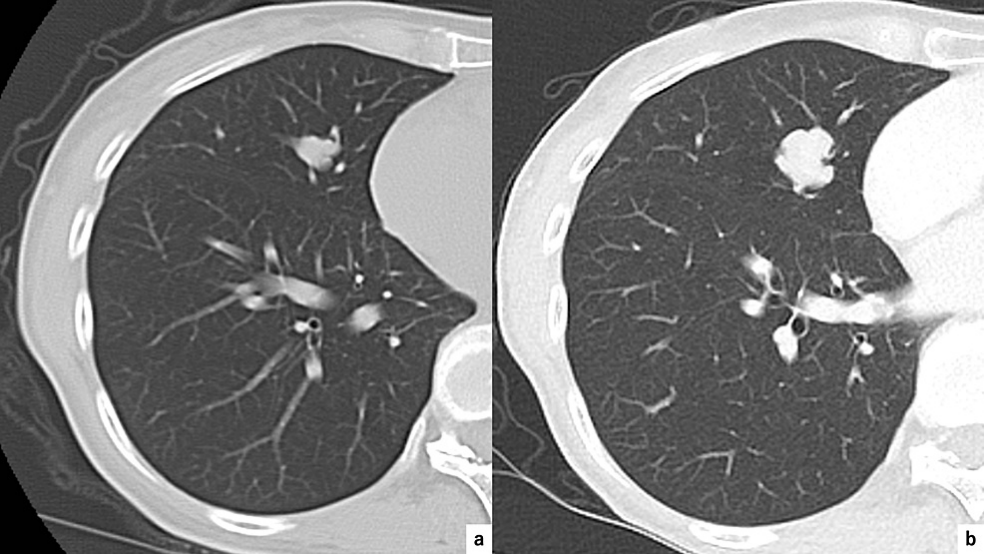
Chest CT findings (a) A chest CT scan taken by a previous doctor 10 years ago shows a nodule in the right middle lobe. (b) The nodule enlarged gradually up to 3.1cm of maximum diameter over 10 years.

**Figure 2:**
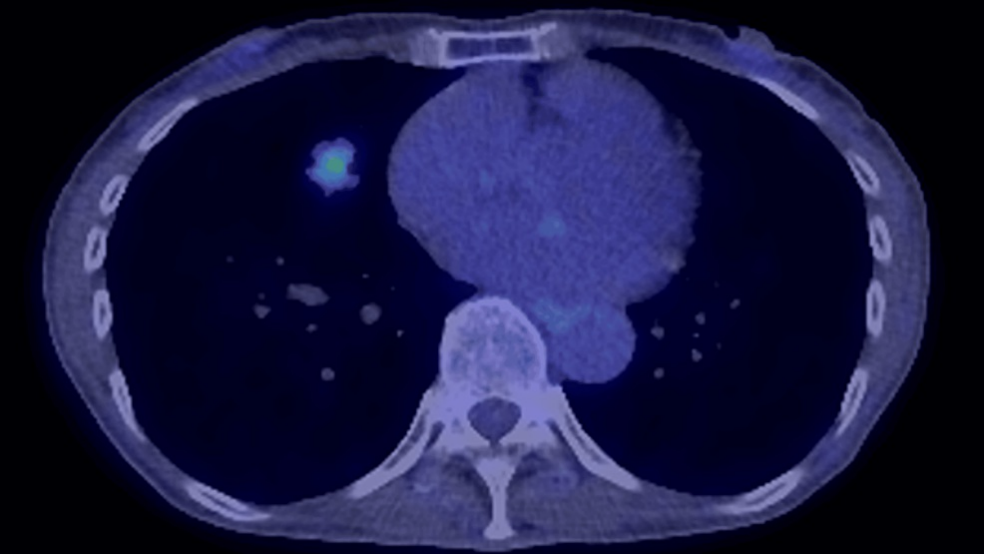
PET-CT findings PET-CT shows mild accumulation of F-18 fluorodeoxyglucose on the tumor (maximum standardized uptake value, 3.17). PET-CT: positron emission tomography-computed tomography

A 63-year-old woman with no notable medical history was referred to our department due to a right lung tumor with progressive enlargement. The patient has no smoking history and no family history, and no physical findings. Chest computed tomography (CT) revealed a well-circumscribed right middle lobe tumor measuring 3.1cm in maximum diameter without calcification and suggestion of pleural invasion. The tumor was located in the proximal part of the right middle lobe. Compared with a previous CT image, which was taken for abnormality of X-ray in medical examination, the tumor had enlarged over 10 years (Figures 1a-1b). However, blood serum levels of tumor markers, such as squamous cell carcinoma-related antigen and carcinoembryonic antigen, were within the normal range. F-18 fluorodeoxyglucose (FDG) positron emission tomography-CT (PET-CT) showed an accumulation of F-18 FDG in the tumor (maximum standardized uptake value, 3.17) (Figure 2). Head contrast-enhanced magnetic resonance imaging showed no lesions. Although the shape of the tumor appeared to be benign, its tendency to grow and the presence of F-18 FDG accumulation could not rule out lung cancer. Therefore, a differential diagnosis of primary lung cancer stage IB (cT2aN0M0) was made. Preoperative biopsies, such as transbronchial biopsy or CT-guided percutaneous needle biopsy, were not performed due to the location of the tumor. However, the case was discussed at a multidisciplinary conference, and surgical resection was thought to be suitable for diagnosis and treatment.

As the tumor was located near the hilum and required a secure surgical margin, a right middle lobectomy was performed via an anterolateral thoracotomy. The postoperative course was uneventful, and the patient was discharged on postoperative day 7.

**Figure 3:**
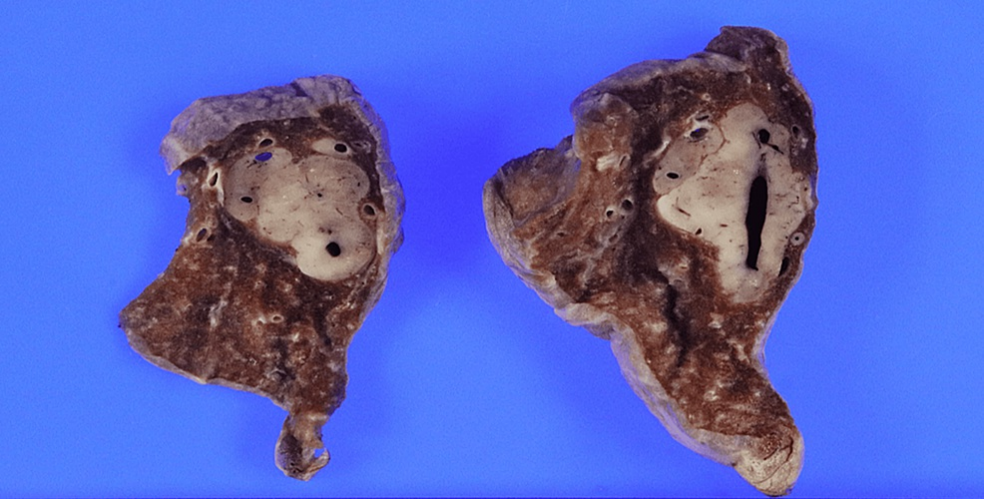
Macroscopic findings Macroscopic findings of the resected tumor showed a well-defined tumor with 3 cm in maximum diameter.

**Figure 4:**
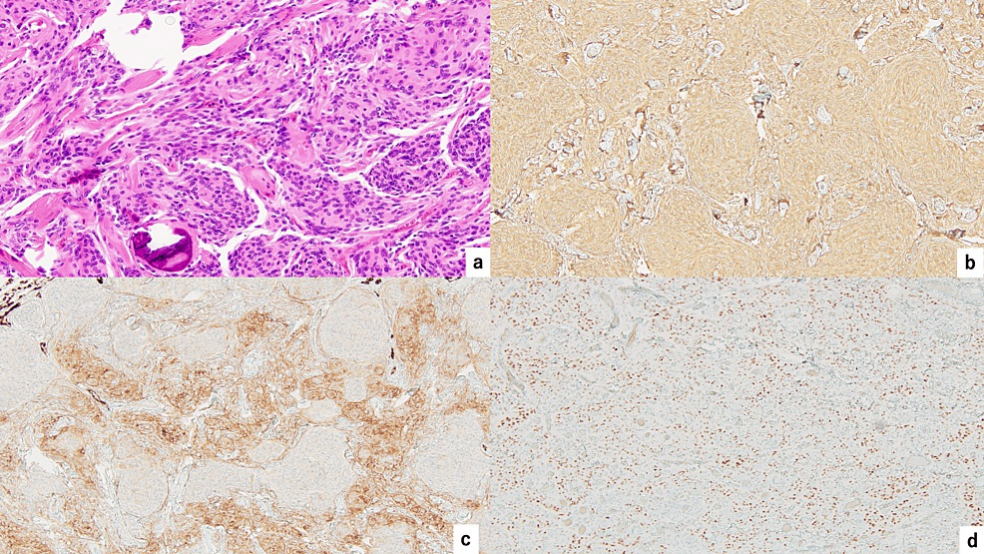
Microscopic findings (a) Hematoxylin and eosin staining. There is proliferation of heteromorphic cells with eosinophilic cytoplasm and analogous round nuclei. Immunochemical staining revealed positivity for (b) vimentin, (c) epithelial membrane antigen (EMA), and (d) progesterone receptor (PgR). Ki-67 proliferative index of the tumor was less than 3%.

Macroscopic findings of the resected tumor showed a well-defined tumor with a maximum diameter of 3 cm (Figure 3). Microscopic findings of the resected tumor stained with hematoxylin and eosin showed the proliferation of the heteromorphic cells with eosinophilic cytoplasm and analogous round nuclei (Figure 4a). Immunohistochemically, the tumor was positive for vimentin, epithelial membrane antigen (EMA), and progesterone receptor (PgR) and negative for chromogranin A, synaptophysin, and thyroid transcription factor-1 (Figures 4b-4d). The Ki-67 index was < 3% and histological and immunohistochemical findings suggested a diagnosis of WHO grade I meningioma. Whole spine contrast-enhanced MRI showed no lesions, like the preoperative head contrast-enhanced MRI. Based on these features, the tumor was diagnosed as PPM.

Thus, the patient was successfully treated surgically, and local and distant recurrences were absent 10 months postoperatively.

## Discussion

Meningiomas often occur in the central nervous system. Primary ectopic meningiomas, account for 1% to 2% of all meningiomas, and typically occur in the head and neck sites, including the nose, paranasal sinus, orbit, oropharynx, and cranium [2]; however, they are uncommon in the lungs. Although they have no established origin, PPMs are thought to originate from ectopic arachnoid cells or differentiated from Schwann cells in the lung [3-6]. Most PPM cases are asymptomatic as they often grow slowly and are usually detected incidentally. On imaging, ruling out the possibility of primary central nervous system meningioma is the key to PPM diagnosis and ruling out metastasis. PPM often presents as well-defined, well-rounded nodules on CT scans, and pulmonary hamartoma, sclerosing pneumocytoma, and carcinoid are included in their differential diagnoses. PPMs might show FDG accumulation; however, it is not a characteristic finding. Therefore, PET-CT has no advantage in the diagnosis of PPM [7-8]. Regarding histopathology, PPMs usually present with spindle-, polygonal-, or ovoid-shaped cells, usually arranged in lobules and whorls, more often without mitoses. Immunohistochemical findings often show positivity for vimentin, EMA, cytokeratin, desmin, and S-100 [9]. Although Masai et al. reported a PPM diagnosed preoperatively by bronchoscopic biopsy, there are limited reports on preoperative PPM diagnosis [10]. Due to the difficulty in securing sample volume by bronchoscopic or percutaneous needle biopsy, a precise preoperative diagnosis of PPMs is difficult [11]. As a benign tumor, PPMs are difficult to diagnose preoperatively because of the possibility of FDG accumulation on PET-CT and the fact that the tumor may increase in size over time, as seen in our case. Therefore, surgical resection is often performed for treatment and diagnosis. The surgical procedure for PPM depends on the tumor location and tumor size; in previous reports, not only wedge resection but lobectomy [12] as well as segmentectomy [9] was also performed for PPMs. In our case, wedge resection was difficult because the tumor had a maximum diameter of 3.1cm, and was located near the hilum. We performed a right middle lobectomy to preserve sufficient surgical margin because malignancy could not be ruled out preoperatively. In surgical treatment for PPMs, complete resection of the tumor is essential, regardless of the surgical procedure. Alfredo et al. reported that PPM appears to have a rather indolent course, with the majority of the patients being disease-free at up to 24 years of follow-up [11]. Nonetheless, there have been few reports of PPMs showing recurrence; however, depending on the karyotype, PPM can present high-grade malignancy. Prayson et al. reported a PPM case with local and distant recurrence within five months after the initial resection [13].

In our case, the Ki-67 positivity rate was < 3%; therefore, the recurrence rate of our PPM case was deemed to be low. However, PPM is a rare disease with limited case reports; thus, the long-term outcome of PPMs remains unknown. Therefore, careful postoperative follow-up is warranted for such cases.

## Conclusions

We report a case of postoperatively diagnosed PPM, which is a rare type of ectopic meningioma. Although malignant PPM is very rare, as in our case, the tumor can show an increasing tendency and may need to be differentiated from lung cancer. There is not yet an established treatment for this rare disease but complete tumor resection is recognized as essential for treatment. Depending on the degree of malignancy, postoperative observation should be performed for PPMs because the possibility of PPM recurrence cannot be ruled out.
